# Berberine-based self-assembly agents with enhanced synergistic antitumor efficacy

**DOI:** 10.3389/fphar.2024.1333087

**Published:** 2024-03-12

**Authors:** Yun Wang, Zhongrui Li, Haili Zhang, Peiye Wu, Yu Zhao, Renshi Li, Chao Han, Lei Wang

**Affiliations:** ^1^ State Key Laboratory of Natural Medicines, Jiangsu Key Laboratory of Bioactive Natural Product Research, School of Traditional Chinese Pharmacy, China Pharmaceutical University, Nanjing, China; ^2^ Department of Rehabilitation, College of Acupuncture and Moxibustion and Massage Health Preservation and Rehabilitation, Nanjing University of Chinese Medicine, Nanjing, China

**Keywords:** carrier-free self-assemblies, natural products, synergistic antitumor, mitochondria-mediated, enhanced efficiency

## Abstract

Tumors are still a major threat to people worldwide. Nanodrug delivery and targeting systems can significantly improve the therapeutic efficacy of chemotherapeutic drugs for antitumor purposes. However, many nanocarriers are likely to exhibit drawbacks such as a complex preparation process, limited drug-loading capacity, untargeted drug release, and toxicity associated with nanocarriers. Therefore, new therapeutic alternatives are urgently needed to develop antitumor drugs. Natural products with abundant scaffold diversity and structural complexity, which are derived from medicinal plants, are important sources of new antitumor drugs. Here, two carrier-free berberine (BBR)-based nanoparticles (NPs) were established to increase the synergistic efficacy of tumor treatment. BBR can interact with glycyrrhetinic acid (GA) and artesunate (ART) to self-assemble BBR-GA and BBR-ART NPs without any nanocarriers, respectively, the formation of which is dominated by electrostatic and hydrophobic interactions. Moreover, BBR-GA NPs could lead to mitochondria-mediated cell apoptosis by regulating mitochondrial fission and dysfunction, while BBR-ART NPs induced ferroptosis in tumor cells. BBR-based NPs have been demonstrated to possess significant tumor targeting and enhanced antitumor properties compared with those of simple monomer mixes both *in vitro* and *in vivo*. These carrier-free self-assemblies based on natural products provide a strategy for synergistic drug delivery and thus offer broad prospects for developing enhanced antitumor drugs.

## 1 Introduction

The incidence and mortality of cancer are increasing, annually worldwide, which suggests that cancer is a risk factor affecting human health (Hanahan, 2022; Siegel et al., 2023). Cancer treatment includes surgery, chemotherapy, radiation therapy, and immunotherapy, while chemotherapy remains the primary treatment in the early and postoperative stages. However, chemotherapy can cause serious adverse reactions in patients, and long-term use of chemical drugs can also lead to problems such as reduced drug sensitivity and resistance (Bagchi et al., 2021). Therefore, developing new antitumor drugs is still a crucial challenge. Natural medicinal plants have positive effects on postoperative adjunctive therapy for certain cancers and are a source of potential antitumor drugs (Islam et al., 2022; Li et al., 2022). In particular, traditional Chinese medicine has made great contributions to the health of people worldwide for thousands of years. Among the 247 anticancer drugs approved by the U.S. Food and Drug Administration between 1981 and 2019, approximately 45% are natural products or natural product derivatives (Newman and Cragg, 2020; Atanasov et al., 2021). Therefore, the use of natural chemical entities with ample structural complexity and scaffold diversity, which are derived from medicinal plants has been, and will continue to be, a prospective method for antitumor drug research.

Various nanocarriers are widely used to load natural molecules to increase therapeutic efficacy and reduce toxicity for the treatment of cancer (Cabral et al., 2020; Ni et al., 2020). Nanotechnology agents, such as liposomes, micelles, carbon materials, and a metal-organic framework, can improve the solubility and prolong the blood circulation time of natural molecules, after which the molecules are actively or passively released into the tumor. However, most nanodrugs face the problems of a low drug loading rate and unsatisfactory drug percolation (Wolfram and Ferrari, 2019). The assembly process of nanodrugs for achieving targeted delivery and targeted drug release is complex and laborious. Therefore, increasing amounts of attention has been given to carrier-free self-assemblies based on pure drugs in the field of nanomedicine development (Huang et al., 2021; Fu et al., 2022; Li et al., 2023). These self-assemblies without any nanocarrier materials are likely formed through electrostatic interactions, hydrophobicity, hydrogen bonding, or π-π stacking. Moreover, carrier-free nanoscale self-assemblies possess a high drug loading capability (even up to 100%), synergistic therapeutic efficacy, and convenient scalable production. The formation of supramolecular structures is facilitated by natural products with unique stereostructures and multiple chiral centers. By transferring and magnifying molecular chirality through self-assembl, natural molecules have been deemed to one of the ideal building blocks for constructing supramolecular nano self-assemblies.

In this work, we successfully prepared two carrier-free self-assemblies, berberine-glycyrrhizic acid nanoparticles (BBR-GA NPs) and berberine-artesunate nanoparticles (BBR-ART NPs), which were used to increase synergistic effects on tumors. BBR (Song et al., 2020) is a famous alkaloid component of *Coptidis rhizoma* and GA (Kowalska and Kalinowska-Lis, 2019) is a representative triterpenoid from *Glycyrrhiza uralensis*. ART (Zhang et al., 2022) is a derivative of artemisinin that is separated from *Artemisia annua*. GA is a pentacyclic triterpene with a rigid hydrophobic skeleton and multiple chiral centers, while ART is a sesquiterpenoid with a flexible alkyl side chain and a rigid hydrophobic skeleton. These special structural properties make it easy for GA and ART to form orderly arranged aggregates with laminar BBR. The diameters of the BBR-ART NPs were larger than those of the BBR-GA NPs, and were dominated by hydrophobic and electrostatic interactions. BBR and GA are released from NPs into the cytosol via the endocytosis of tumor cells, after which BBR specifically targets mitochondria. After intravenous injection, the assembled BBR-GA NPs may be passively targeted to tumor tissue where they accumulate. BBR-GA NPs could lead to mitochondria-mediated cell apoptosis by regulating mitochondrial fission and dysfunction. In contrast, BBR-ART NPs induced ferroptosis in PANC-1 cells *in vitro* and *in vivo*. BBR-based NPs enabled synergistic antitumor and sensibilized single drug-based antineoplastic effects. This study provides an effective strategy to enhance the antitumor efficacy of carrier-free self-assemblies based on natural products.

## 2 Materials and methods

### 2.1 Reagents

BBR, GA, and ART were purchased from Shanghai Aladdin Reagent Co., Ltd., China. An Annexin V-FITC/7ADD apoptosis detection kit, and a bicinchoninic acid protein assay kit were purchased from Jiangsu KeyGen BioTech. Corp., Ltd. (Nanjing, China). DMEM, bovine serum albumin, fetal bovine serum, 2′,7′-dichlorodihydrofluorescein diacetate, BODIPY lipid peroxidation fluorescent probe, and PBS were purchased from Invitrogen Life Technologies (Carlsbad, USA). Terminal deoxynucleotidyl transferase (TdT)-mediated dUTP nick end labeling apoptosis staining kit and Ki67 cell proliferation detection kit were purchased from Vazyme Biotech, Nanjing, China. Antibodies against FIS1, OPA1, Bcl2, Bax, cleaved caspase 3, xCT, p53, GPX4, HO-1, and GAPDH were purchased from Cell Signaling Technology (Beverly, MA, USA). Other solvents used in this study were of analytical grade, and distilled deionized water was used.

### 2.2 Preparation and characterization of BBR-based NPs

BBR-based NPs were prepared by simple agitation. Briefly, GA or ART aqueous solutions (0.1 mM) were mixed with hydrochloric acid (BBR) aqueous solution (0.1 mM) in the ratio of 1:1. This mixture was stirred for 12 h at 24°C. The mixture was dialyzed against deionized water for 12 h (M_W_ = 7,000 Da), after which the unencapsulated GA, ART, and BBR were removed. Then, the obtained BBR-GA NPs or BBR-ART NPs were resuspended in phosphoric acid buffer solution and stored at 4°C.

The morphological characteristics of the BBR-based NPs were determined by HT7700 Transmission Electron Microscopy. A nanoparticle tracking analysis system (Zeta View, Meer Busch, Germany) was used to determine the size of the NPs, and a Zetasizer Nano ZS90 (Malvern, Worcestershire, UK) was used to determine the zeta potential. Ultraviolet−visible (UV−vis, 200–500 nm) and Fourier transform infrared (FT-IR, 4,000 to 400 cm^−1^ with the KBr method) spectra of the samples were obtained by a UV-2450 UV−vis spectrophotometer (Shimadzu, Japan) and a Fourier transform infrared spectrometer (Bruker, Germany), respectively. The fluorescence spectra of the samples were measured on an RF-6000 Spectro fluorophotometer (Shimadzu, Japan) in the range of 400–700 nm. ^1^H-NMR and ROESY spectra were obtained with a Bruker AVIII-600 NMR spectrometer (^1^H: 600 MHz) (Bruker, Germany), with tetramethylsilane (TMS) serving as an internal standard. Chemical shift values (*δ*) are given in parts per million (ppm). The dialysis method was used to investigate the release of BBR from NPs. The NPs (2 mL) were soaked in PBS (35 mL) containing 0.1% tween 80. The NPs in PBS at pH 7.4 or 5.5 were collected at the indicated time points. The release of BBR was analyzed by using a microplate reader (350 nm wavelength), and the release rate was calculated.

### 2.3 Cellular uptake and subcellular localization of BBR-GA NPs

A549 cells (3 × 10^3^ cells per well) were seeded in glass bottom 96-well plates. When the cells were adherent, they were treated with BBR-GA NPs (at encapsulated BBR concentration of 30 μM) for 0, 1, 2, 4, or 6 h. Cells were cleaned three times. Fluorescence images were captured by using a high-content screening system. To study the subcellular location of BBR-GA NPs, A549 cells (3 × 10^3^ cells per well) were seeded in glass bottom 96-well plates. Cells were treated with NPs (30 μM BBR equiv.) for 2 h or 8 h, after which the cells were washed three times with PBS. Cells were further incubated with 1.0 μM Lyso-Tracker Red or Mito-Tracker for 15 min, and the nuclei were stained with 10 μM DAPI for 15 min. Fluorescence images were taken using an HCS system.

### 2.4 *In vitro* antitumor efficacy

MTT assays were applied to determine the cytotoxicity of the BBR-based NPs. A549, MCF-7, and PANC-1 cells (3.0 × 10^3^ cells) were seeded in 96-well plates. Cells were incubated with different dosage forms for 24 h. Then, 10 μL of 5 mg mL^−1^ MTT solution in PBS was added to each well. After another 4 h of incubation, 150 μL of DMSO was added to each well to dissolve the generated formazan. After shaking for 15 min, the absorbance of obtained formazan was measured at 570 nm by using a SpectraMax Plus384 microplate reader (Molecular Devices, CA, USA). Clonogenic ability assay was conducted to evaluate the inhibitory effect of BBR-ART NPs on the proliferation of PANC-1 cells. Cells (1.0 × 10^3^ cells per well) were seeded in 6-well plates. Cells were treated with different dosage forms of BBR-ART NPs or 0.1% DMSO for 6 d. The cells in the plates were then stained with crystal violet and imaged. Apoptosis induction by BBR-GA or BBR-ART NPs was measured by using Annexin V-FITC/7ADD double staining assays. A549, MCF-7, or PANC-1 cells (2 × 10^5^ cells per well) were treated with different dosage forms of BBR-based NPs. After another 24 or 48 h of incubation, cells were washed with PBS, digested with trypsin, and stained in binding buffer containing annexin V-FITC and 7ADD for 15 min. Flow cytometry was used to analyze the cells. The mitochondrial membrane potential was measured using a JC-1 assay. After A549 cells were treated with different dosage forms of JC-1, 500 μL JC-1 dye staining solution, was added, and the cells were incubated in the dark at 37°C for 25 min. Cells were washed twice and detected by flow cytometry. Intracellular reactive oxygen species (ROS) production or lipid peroxidation induced by NPs. A549 or PANC-1 cells were cultured with different, treatment regimens and washed with PBS. Then cells were treated with DCFH-DA or BODIP for 30 min at 37°C. Flow cytometry and an HCS system were used to measure the relative ROS or lipid peroxidation levels.

### 2.5 Western blotting

Western blotting was used to determine the protein expression of FIS1, OPA1, Bcl2, Bax, cleaved caspase 3, xCT, p53, GPX4, and HO-1 in cell lysates. GAPDH was used as the reference protein. Equal amounts of proteins from the total cell lysates were separated via sodium dodecyl sulfate 10% polyacrylamide gel electrophoresis. The proteins were wet-transferred to a polyvinylidene fluoride membrane in the presence of an electric field and blotted with primary antibodies specific for FIS1, OPA1, Bcl2, Bax, cleaved caspase 3, xCT, p53, GPX4, HO-1, and GAPDH, which were used as the internal standards, overnight. Samples were then probed with secondary antibodies for 2 h at 37°C. The prepared protein samples were tested using the Chemi DOC^TM^ XRS+ system (Bio-Rad Laboratories, Hercules, CA, USA).

### 2.6 Zebrafish xenograft tumor model

All experiments were approved by the Animal Ethical Committee of China Pharmaceutical University, and the handling procedures were performed on the basis of the National Institutes of Health of Experimental Animals. Wild type zebrafish were purchased from Nanjing Xinjia Medical Technology Co., Ltd. Zebrafish were fed in E3 embryo media at 28°C. A549 cells were incubated with 10 μg mL^−1^ CM-DiI solution (5% DMSO/water) until fluorescence was observed. The labeled cells were microinjected into the yolk space of 48 hpf wild-type zebrafish embryos (400 cells per embryo). Zebrafish xenograft tumor model was established and cultured in a 34°C light incubator (light 14 h, dark 10 h). The tumor-bearing zebrafish were randomly divided into different groups (3 or 6 zebrafish/group). The zebrafish were incubated with BBR + GA or BBR-GA NPs (5 mM BBR equiv.). Fluorescence images were recorded using a fluorescence microscope at 0, 30, and 60 min for detection of tumor targeting. Next, to evaluate anti-tumor efficacy of BBR-GA NPs, tumor-bearing zebrafish were incubated with PBS, BBR + GA, or BBR-GA NPs (5 mM BBR equiv.). Fluorescence images were recorded using a fluorescence microscope at 0 and 4 days for determination of antitumor effects.

### 2.7 *In vivo* antitumor efficacy and safety

Five-week-old BALB/c nude mice (GemPharmatech Co., Ltd, Nanjing, China) were selected and injected with A549 or PANC-1 cells (4.0 × 10^7^ cells per mouse) into the right lower limb region. When the tumors reached a size of 100 mm^3^, the tumor-bearing mice were randomly divided into different groups (7 mice/group). Then, the mice were intravenously administered different dosage forms every 2 days. Throughout the experiments, the bodies of the mice were observed. Tumor volume (mm^3^) was measured using calipers every 2 days and the tumor volume was calculated with the following formula (length × width^2^ × 0.5). After 14 days of treatment, the mice were euthanized and sacrificed, and the tumor tissues and major organs were harvested for hematoxylin and eosin (H&E) staining, TUNEL assays and immunohistochemical assays. To evaluate the safety of BBR-based NPs *in vivo*, the serum levels of blood urea nitrogen (BUN), creatinine (CRE), aspartate transaminase (AST), alanine transaminase (ALT) were measured by using the corresponding assay kits (KeyGen, China).

### 2.8 Statistical analysis

All experiments were repeated at least in triplicate and the data are expressed as the mean ± standard deviation (SD). Results were analyzed by one-way analysis of variance (ANOVA) using GraphPad Prism 6.0 (GraphPad Inc., San Diego, CA). A *p*-value less than 0.05 was considered to indicate a significant difference. *p < 0.05, **p < 0.01, ***p < 0.001, and ****p < 0.0001 were considered to indicate statistical significance.

## 3 Results and discussion

### 3.1 Preparation and characterization of BBR-GA and BBR-ART NPs

**FIGURE 1:**
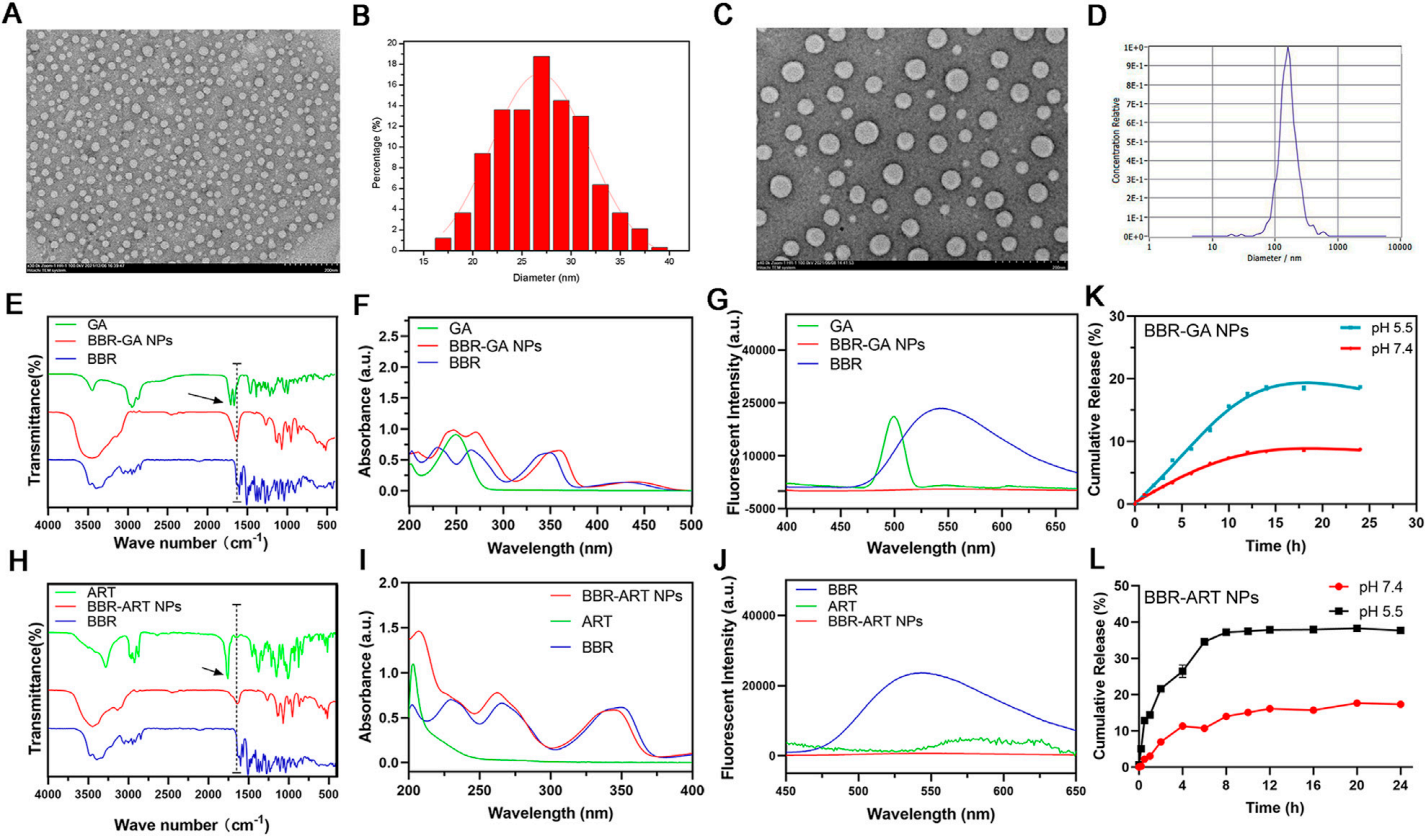
Characterization of BBR-based NPs. **(A)** TEM images of BBR-GA NPs. **(B)** The size distribution of BBR-GA NPs as determined by TEM images. **(C)** TEM images of BBR-ART NPs. **(D)** Size distribution of BBR-ART NPs in aqueous solution. **(E)** IR, **(F)** UV−vis, and **(G)** emission spectra of BBR-GA NPs and their monomers. **(H)** IR, **(I)** UV−vis, and **(J)** emission spectra of BBR-ART NPs and their monomers. *In vitro* BBR release from BBR-GA **(K)** and BBR-ART NPs **(L)**. Data are presented as the mean ± SD, *n* = 3.

BBR and GA/ART could self-assemble into NPs in aqueous solution. Several characteristic constants of the self-assemblies were examined. TEM (Figures 1A, B) revealed that the morphology of BBR-GA was uniform, and the particle size was approximately 26.5 nm. The self-assembled form of the BBR-ART system was similar to that of BBR-GA NPs. These NPs were approximately 137.9 nm in diameter, which waw larger than that of the BBR-GA NPs (Figures 1C, D). The zeta potentials of BBR-GA NPs and BBR-ART NPs were −24.5 ± 1.1 and −18.4 ± 1.0 mV (Supplementary Figure S1). Moreover, all of the BBR-GA NPs and BBR-ART NPs exhibited the Tyndall effect in aqueous solution (Supplementary Figure S2). The stability of BBR-GA NPs and BBR-ART NPs in physiological condition (Supplementary Figure S3). Next, IR, UV, and fluorescence emission spectra were used to measure the spectroscopic properties of BBR-based self-assemblies. In IR spectroscopy, GA showed strong absorption peaks at 1706 and 1,664 cm^−1^ (corresponding to the C=O stretching peaks of GA, Figure 1E). However, these peaks became single peak that shifted to a lower wavenumber (1,640 cm^−1^) in the BBR-GA NPs. BBR-ART NPs exihibited the same IR spectroscopic properties (Figure 1H). This result indicated that electrostatic interactions formed between the carboxyl group of GA/ART and ammonium ion of BBR. The UV–vis spectrum (Figure 1F) of BBR-GA NPs contained three typical absorption bands for GA and BBR (228, 262, and 344 nm). Similar to BBR-GA NPs, BBR-ART also exhibited three typical absorption bands (209, 260, and 347 nm), which are signals of ART and BBR, respectively (Figure 1I). BBR-based NPs exhibited the characteristic UV peaks of their monomers. Furthermore, the fluorescence characteristics of the BBR-based NPs were studied. BBR emitted obvious fluorescence at 550 nm (Figures 1G, J). After being assembled, NPs showed no significant fluorescence absorption. This difference might be due to fluorescence quenching resulting from the energy transfer effect after self-assembly. The *in vitro* profiles of BBR released from NPs in pH 5.5 and pH 7.4 solutions were generated based on incubation times (Figures 1K, L). NPs displayed a higher BBR release rate at pH 5.5 than at pH 7.4, indicating that BBR was released from NPs to a greater extent in an acidic microenvironment than in a neutral environment.

**FIGURE 2:**
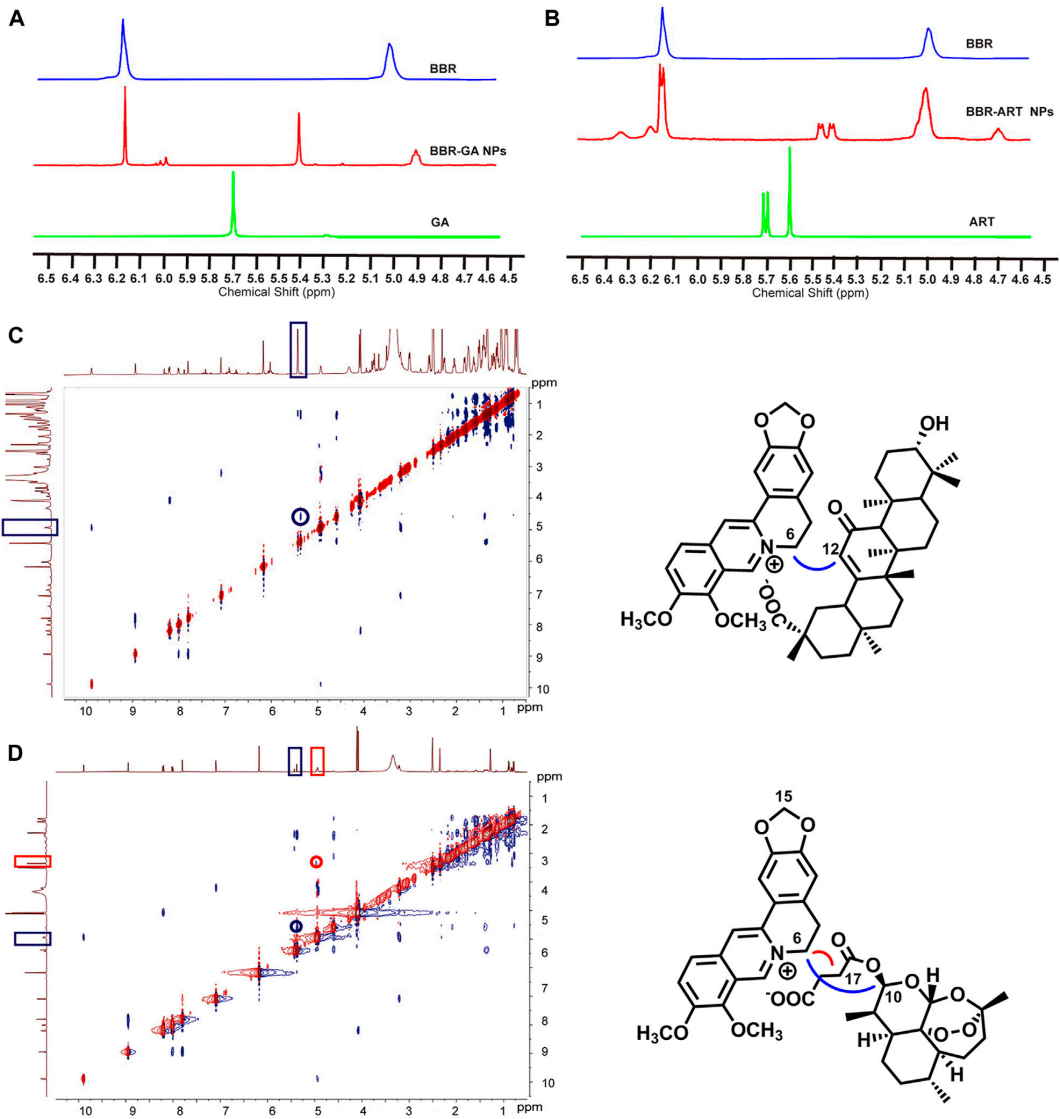
Nuclear magnetic resonance spectra of BBR-based NPs. **(A)** ^1^H NMR spectra of BBR-GA NPs and their monomers (600 MHz, DMSO-*d*_6_). **(B)** ^1^H NMR spectra of BBR-ART NPs and their monomers (600 MHz, DMSO-*d*_6_). ROESY correlation spectra of BBR-GA **(C)** and BBR-ART NPs **(D)**.

Moreover, ^1^H-NMR and ROESY spectra were also obtained to confirm the formation of self-assembled BBR-based NPs. For BBR-GA NPs, the chemical shift of H-6 in BBR decreased from 5.02 to 4.93 ppm, while the chemical shift of H-12 of GA decreased from 5.77 to 5.43 ppm (Figure 2A). Correspondingly, the ROESY spectrum of BBR-GA NPs showed strong interactions between H-6 of BBR and H-12 of GA (Figure 2C), which suggests that the distances between these two hydrogen atoms were closer to each other (less than 5 Å). Combined with the previous IR spectra of BBR-GA NPs, these results further confirmed that the formation of self-assembled NPs was driven by electrostatic interactions between carboxyl group of GA and ammonium ion of BBR. For the BBR-ART NPs, the chemical shift of H-15 of BBR did not change, while the peak shape of these hydrogen atoms changed from singlet to doublet (Figure 2B). The chemical shift of H-17 in ART decreased from 5.61 to 4.75 ppm, while the chemical shift of H-10 in ART decreased from 5.71 to 5.43 ppm. Observably, the peak shape of H-10 for ART changed from a doublet to a double doublet. Next, in the ROESY spectrum of BBR-ART NPs, strong interactions between H-6 of BBR and H-12/-10 of ART were observed (Figure 2D). Overall, electrostatic interactions drove the formation of a one-dimensional BBR-based complex: a parallel conformation between BBR and GA, and a staggered conformation between BBR and ART. Then, hydrophobic interactions drove the formation of three-dimensional BBR-based NPs. The formation processes of these two BBR-based NPs were different. Previous results in which the diameters of BBR-ART NPs were larger than those of the BBR-GA NPs might confirm these findings. In conclusion, BBR-based NPs were validated to be self-assembled nanoplatforms in various assays.

### 3.2 Cellular uptake and intracellular localization of BBR-GA NPs *in vitro*

**FIGURE 3:**
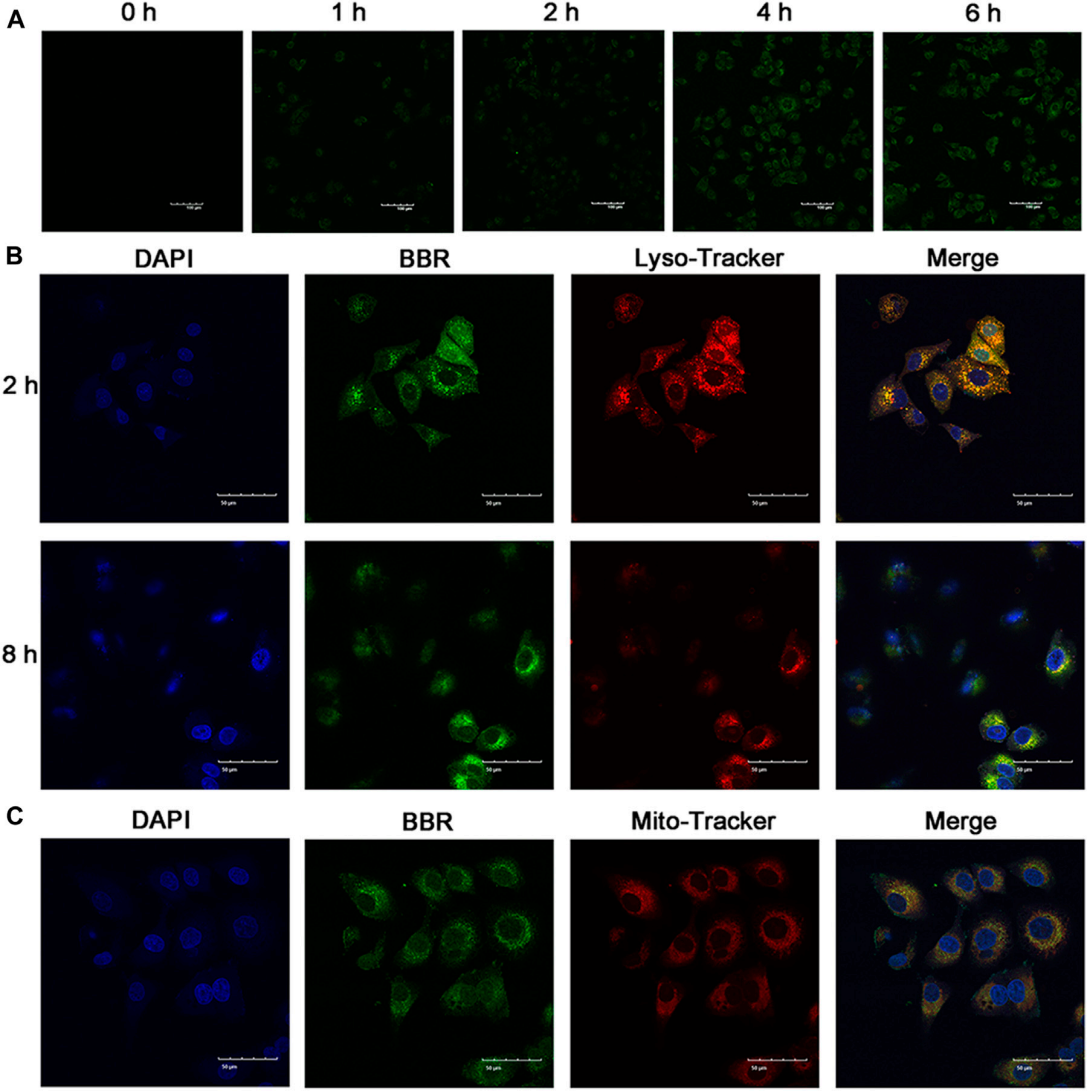
Cellular uptake and subcellular distribution *in vitro*. **(A)** Fluorescence images of A549 cells after treatment for 0-6 h with BBR-GA NPs (30 μM BBR equivalent). Scale bar = 100 μm. **(B)** Fluorescence images of BBR-GA NPs with lysosomal localization (30 μM BBR equivalent) in A549 cells after treatment for 2 and 8 h. Scale bar = 50 μm. **(C)** Fluorescence images of BBR-mediated targeting of mitochondria (30 μM BBR equivalent) in A549 cells after treatment for 8 h. Scale bar = 50 μm.

Cellular uptake of NPs has a significant influence on their antitumor effect. Thus, to investigate the cellular uptake of BBR-GA NPs, human A549 lung cancer cells were used as a cell model. To study the cellular uptake of BBR-GA NPs, A549 cells were incubated with NPs, and the fluorescence intensity of BBR (green) was used to determine the quantity of BBR-GA NPs taken up. The green fluorescence intensity increased over time within 6 hours (Figure 3A). After endocytosis, the subcellular localization of the BBR-GA NPs was analyzed. To explore the mitochondrial targeting of BBR after the lysosomal capture of BBR-GA NPs in A549 cells, fluorescence imaging assays were performed. The number of lysosomes was determined using LysoTracker. BBR (green fluorescence) was clearly visualized after 2 h (Figure 3B). Orange fluorescence which was green (BBR) combined with red (lysosome) fluorescence, indicated that BBR-GA NPs were trapped by lysosomes. After 8 h of incubation, weak orange fluorescence demonstrated that BBR-GA NPs had escaped from lysosomes. After lysosomal escape, BBR was proven to target mitochondria. DAPI (blue) was used for nuclear staining and Mito-Tracker Red was used for mitochondrial staining. The bright orange fluorescence (Figure 3C) indicated the mitochondrial targeting of BBR. There was a negative large transmembrane potential in the mitochondria, which might drive the positively charged BBR to the mitochondria. Therefore, BBR-GA NPs were endocytosed into tumor cells where they rapidly released BBR and GA after lysosomal escape. Then, BBR targets mitochondria and GA is exposed to the cytoplasm.

### 3.3 *In vitro* antitumor effects of BBR-based NPs

**FIGURE 4:**
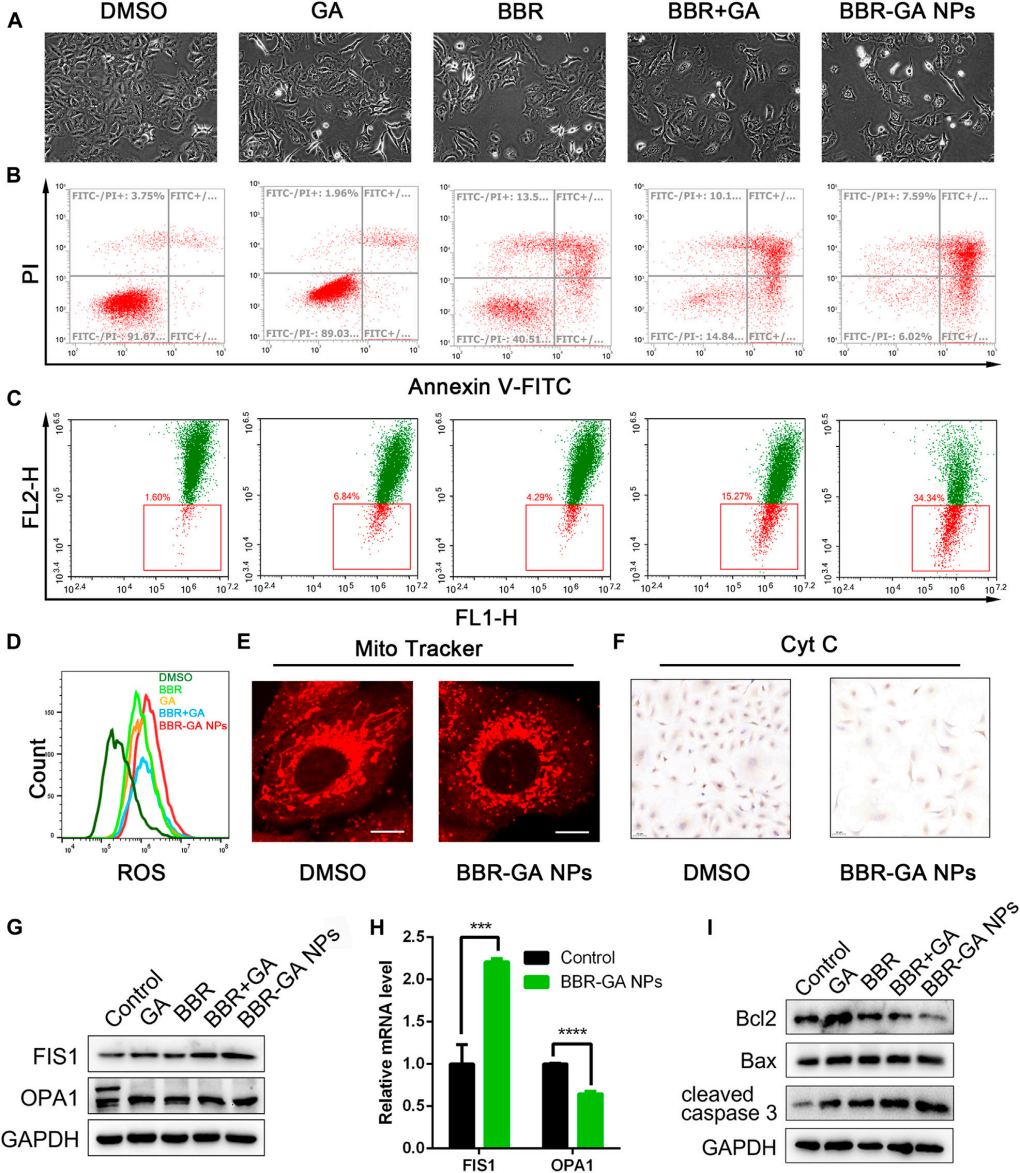
*In vitro* proapoptotic activity of BBR-GA NPs. **(A)** Cellular morphology of A549 cells after treatment with various formulations for 48 h. **(B)** Cell apoptosis after treatment with various formulations determined by annexin V-FITC/7ADD staining. **(C)** ΔΨm of A549 cells after incubation with various formulations determined by JC-1 staining. **(D)** Analysis of ROS production in A549 cells using flow cytometry. **(E)** Confocal microscopy images of BBR-GA NPs-induced mitochondrial fission as determined via MitoTracker staining. Scale bars: 20 μm. **(F)** Immunohistochemical staining images of cytochrome c translocated from mitochondria to cytosol in A549 cells after treatment with BBR-GA NPs. **(G)** The expression of FIS1 and OPA1 in A549 cells after treatment with various formulations was determined by Western blot analysis. **(H)** Relative mRNA levels of FIS1 and OPA1 in A549 cells after treatment with BBR-GA NPs determined by Q-PCR analysis. **(I)** The expression of Bcl2, Bax, and cleaved caspase 3 in A549 cells after treatment with various formulations by Western blot analysis.

To explore the cytotoxicity of the BBR-GA self-assemblies, MTT assays were used with A549 cells. The IC_50_ value of BBR-GA NPs was 60.5 ± 0.13 μM (Supplementary Figure S4). BBR-GA NPs at high concentrations (>50 μM) had higher cytotoxic activity than the GA, BBR, or BBR + GA groups. In contrast, the IC_50_ value was lower when human lung bronchial epithelial BEAS-2B cells were treated with BBR-GA NPs (IC_50_ > 100 μM), indicating that self-assemblies produced stronger toxicity particularly in tumor cells. Compared to those in the other groups, BBR-GA NPs caused the cells to become shrunken and sparse (Figure 4A). Similar results were obtained for the apoptosis-inducing effect. The total apoptosis rate induced in the BBR-GA NPs group was 86.4%, which was higher than that in the BBR + GA group (11.3%), BBR group (40.4%), and GA group (77.4%) (Figure 4B; Supplementary Figure S4). Mitochondria-mediated apoptosis is regarded as the major mode of cell death in cancer therapy (Harrington et al., 2023; Peng et al., 2023), and is characterized by a decrease in the mitochondrial membrane potential (ΔΨm), triggering the production of ROS and the release of proapoptotic factors (such as caspase-3). The ability of BBR-GA NPs to reduce the ΔΨm was measured in A549 cells by using the lipophilic dye JC-1. The ΔΨm of cells in all the administration groups was reduced, compared with that of cells in the control group, and BBR-GA NPs were the most effective decreasing the ΔΨm to 65.7% (Figure 4C; Supplementary Figure S4). Next, the cell-permeable fluorophore DCFH-DA was used to assess the production of intracellular ROS. The ROS levels in the BBR-GA group were 1.5, 1.3, and 1.1 times higher than those in GA, BBR, and BBR + GA groups, respectively (Figure 4D; Supplementary Figure S5). Next, MitoTracker Red staining demonstrated that mitochondria of A549 cells showed obvious morphological changes from an oval shape to a truncated and fragmented shape following BBR-GA NPs treatment (Figure 4E). The above results suggested mitochondrial dysfunction could trigger the release of cytochrome c from mitochondria to cytoplasm. BBR-GA NPs significantly reduced the release of cytochrome c by means of streptavidin-peroxidase immunohistochemistry (Figure 4F). Next, Western blotting and Q-PCR were used to evaluate the balance of mitochondrial dynamics. Mitochondria undergo fission when cells are under metabolic or environmental stress (Kraus et al., 2021). Fission 1 (FIS1) is a mitochondrial fission-associated factor, while optic atrophy 1 (OPA1) is related to mitochondrial fusion (Rodrigues and Ferraz, 2020). The expression of FIS1 increased in BBR-GA NPs-treated cells (Figures 4G, H; Supplementary Figure S6). The ratio of L-OPA1 (long isoform of OPA1) to S-OPA1 (short isoform of OPA1) in cells dramatically decreased upon treatment with BBR-GA NPs. Moreover, the ratio of Bax (proapoptotic protein) to Bcl-2 (anti-apoptotic protein), which can initiate the cascade of caspases (Walensky, 2019), was further measured. Similarly, the Bax/Bcl-2 ratios in the BBR-GA NPs group were 1.4-fold, 1.6-fold, and 3.0-fold higher than those in the BBR + GA, BBR, and GA groups, respectively (Figure 4I; Supplementary Figure S7). Consistently, BBR-GA NPs group enhanced cleaved caspase-3 activity by 1.1-fold, 1.4-fold, and 2.0-fold compared to that in the BBR + GA, BBR, and GA groups, respectively. Collectively, BBR-GA NPs reduced imbalance of mitochondrial dynamics to promote mitochondrial fission, thereby leading to mitochondria-mediated cell apoptosis.

**FIGURE 5:**
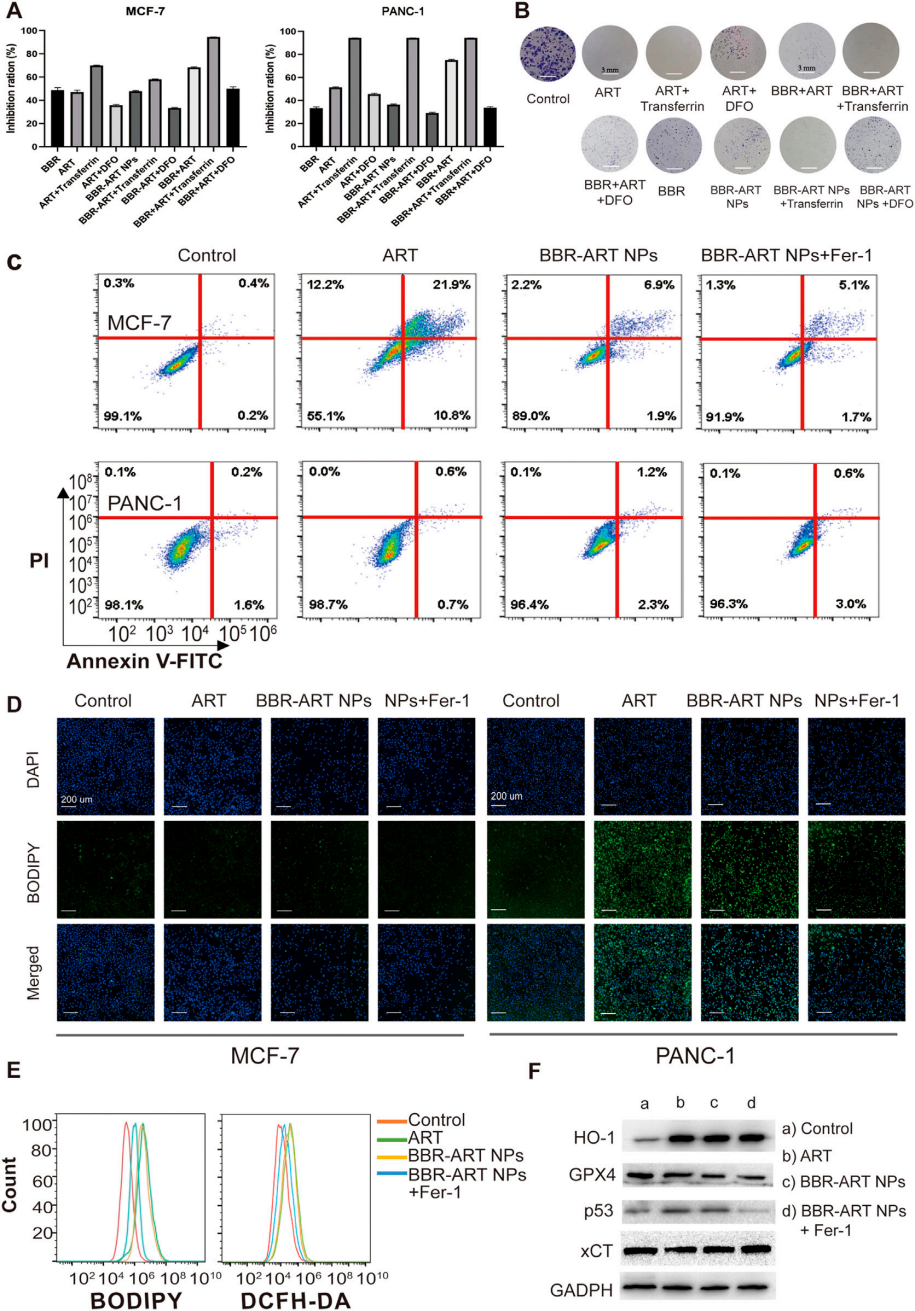
*In vitro* pro-ferroptosis activity of BBR-ART NPs. **(A)** MTT analysis of MCF-7 and PANC-1 cells after exposure to various formulations. The data are presented as the mean ± SD, *n* = 3. **(B)** The clonogenic ability of PANC-1 cells after treatment with various formulations. **(C)** The apoptotic rate of MCF-7 and PANC-1 cells after treatment with various formulations for 24 h determined by annexin V-FITC/7ADD staining. **(D)** Fluorescence microscopic images of lipid ROS in MCF-7 and PANC-1 cells after BODIPY staining. Scale bar = 200 μm. **(E)** Analysis of lipid ROS levels in PANC-1 cells by BODIPY staining and total ROS levels by DCFH-DA staining. **(F)** The expression of HO-1, GPX4, p53, and xCT in PANC-1 cells was determined by Western blot analysis.

Ferroptosis is a type of cell death characterized by the accumulation of lipid peroxidation products and iron, and is different from apoptosis, autophagy, and cell necrosis (Lei et al., 2022; Liang et al., 2022). Artemisinin derivatives can induce ferroptosis in the treatment of cancer (Zhu et al., 2020; Hu et al., 2022). BBR-ART NPs (50 µM BBR equiv.) induced approximately 36.5% cell death in PANC-1 cells (Figure 5A). Increasing lysosomal free iron by cotreatment with iron-saturated, diferric holo-transferrin significantly increased cell death (94.2%). However, coaddition of the lysosomal iron chelator deferoxamine mesylate (DFO) blocked cell death (29.1%), indicating that BBR-ART NPs-induced ferroptosis in PANC-1 cells. Control MCF-7 cells were insensitive to all conditions. Colony formation assays were subsequently performed to determine the effects of BBR-ART NPs on proliferation. BBR-ART NPs reduced clonogenic growth of PANC-1 cells after 6 days of treatment (Figure 5B; Supplementary Figure S8). Consistent with MTT results, the clonogenic growth of cells was amplified by cotreatment with transferrin and rescued by coaddition of DFO. Annexin V-FITC/PI apoptosis detection showed BBR-ART NPs and BBR-ART NPs + Fer-1 groups (ferrostatin-1, a ferroptosis inhibitor) exhibited very little apoptosis in PANC-1 cells compared with MCF-7 cells (Figure 5C), further highlighting the effect of NPs-mediated ferroptosis in PANC-1 cells. Iron-dependent ROS generation during ferroptosis is the central stressor for cellular death (Wang Y. et al., 2021). A BODIPY probe was used to measure intracellular lipid peroxidation. Green fluorescence (with oxidized BODIPY) was clearly observed in PANC-1 cells compared to MCF-7 cells (Figure 5D). The fluorescence intensities of BBR-ART NPs in PANC-1 cells were approximately 1.2 and 2.0 times higher than those in the ART and BBR-ART NPs + Fer-1 groups, respectively (Supplementary Figure S9). Fer-1 was shown to inhibit lipid peroxidation during ferroptotic cell death. Similarly, flow cytometry yielded similar (Figure 5E; Supplementary Figure S10). Next, Western blot analysis was used to evaluate the expression of ferroptosis-related factors: heme oxygenase-1 (HO-1, which catalyzes the catabolism of heme to ferrous) and glutathione peroxidase 4 (GPX4, a negative regulator). HO-1 expression increased and GPX4 expression decreased in response to BBR-ART NPs treatment (Figure 5F; Supplementary Figure S11), indicating activation of ferroptosis. Moreover, BBR-ART NPs could also decrease the expression of xCT and increase the expression of p53. In general, BBR-ART NPs exhibited antitumor activity in PANC-1 cells by inducing ferroptosis.

### 3.4 *In vivo* antitumor effects of BBR-based NPs

**FIGURE 6:**
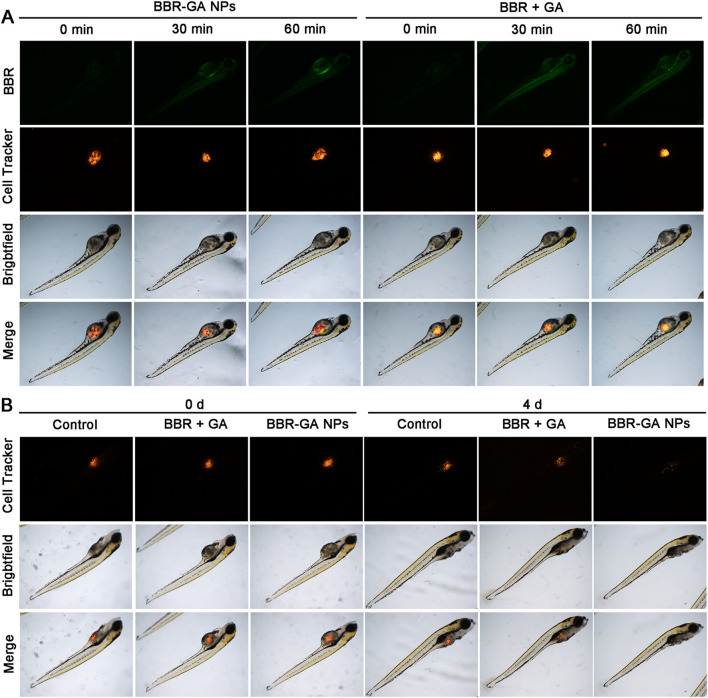
*In vivo* biodistribution and therapeutic efficacy of BBR-GA NPs in A549 tumor-bearing zebrafish. **(A)** Fluorescence images of zebrafish at 0, 30 and 60 min after treatment with BBR-GA NPs or BBR + GA. **(B)** Fluorescence images of zebrafish at 0 and 4 days after treatment with BBR-GA NPs or BBR + GA.

As expected, BBR-based NPs showed more favorable tumor targeting than the other monomers due to the enhanced permeability and retention (EPR) effect. BBR fluorescence can be used to detect the biodistribution of BBR-GA NPs in A549 tumor-bearing zebrafish. Zebrafish (Astell and Sieger, 2020) are becoming a suitable model for testing tumor targeting of NPs because of the transparency of zebrafish embryos, which enables the visualization of fluorescently labeled cancer cells and NPs through their body wall in real time. In addition, zebrafishes exhibit 87% homology with the human genome (Wang X. et al., 2021; Wu et al., 2021), which can be used to characterize the anticancer effects of NPs. A xenograft model was established by microinjecting Cell-Tracker™ CM-Dil labeled A549 cells into the yolk sacs of zebrafish embryos. BBR signals (green) of BBR + GA group were clearly observed in zebrafish after 30 min of treatment and the green fluorescence intensity was always distributed throughout the body at 60 min (Figure 6A). However, BBR-GA group showed obvious drug accumulation at the tumor site, with green fluorescence approaching the yolk sacs of zebrafish at the treatment time. These data supported the ability of BBR-GA NPs to target tumors *in vivo*. Next, the *in vivo* antitumor activity of BBR-GA NPs was studied in A549 tumor-bearing zebrafish. After 4 days of treatment, BBR-GA NPs and BBR + GA groups all exhibited reduced fluorescence intensity in the zebrafish (Figure 6B). Similarly, compared with those in the control group, the tumor inhibition rates in the BBR-GA NPs and BBR + GA groups were 86.4% and 69.6%, respectively (Supplementary Figure S12). BBR-GA NPs inhibited tumor growth more than the simple monomer mixture in zebrafish model.

**FIGURE 7:**
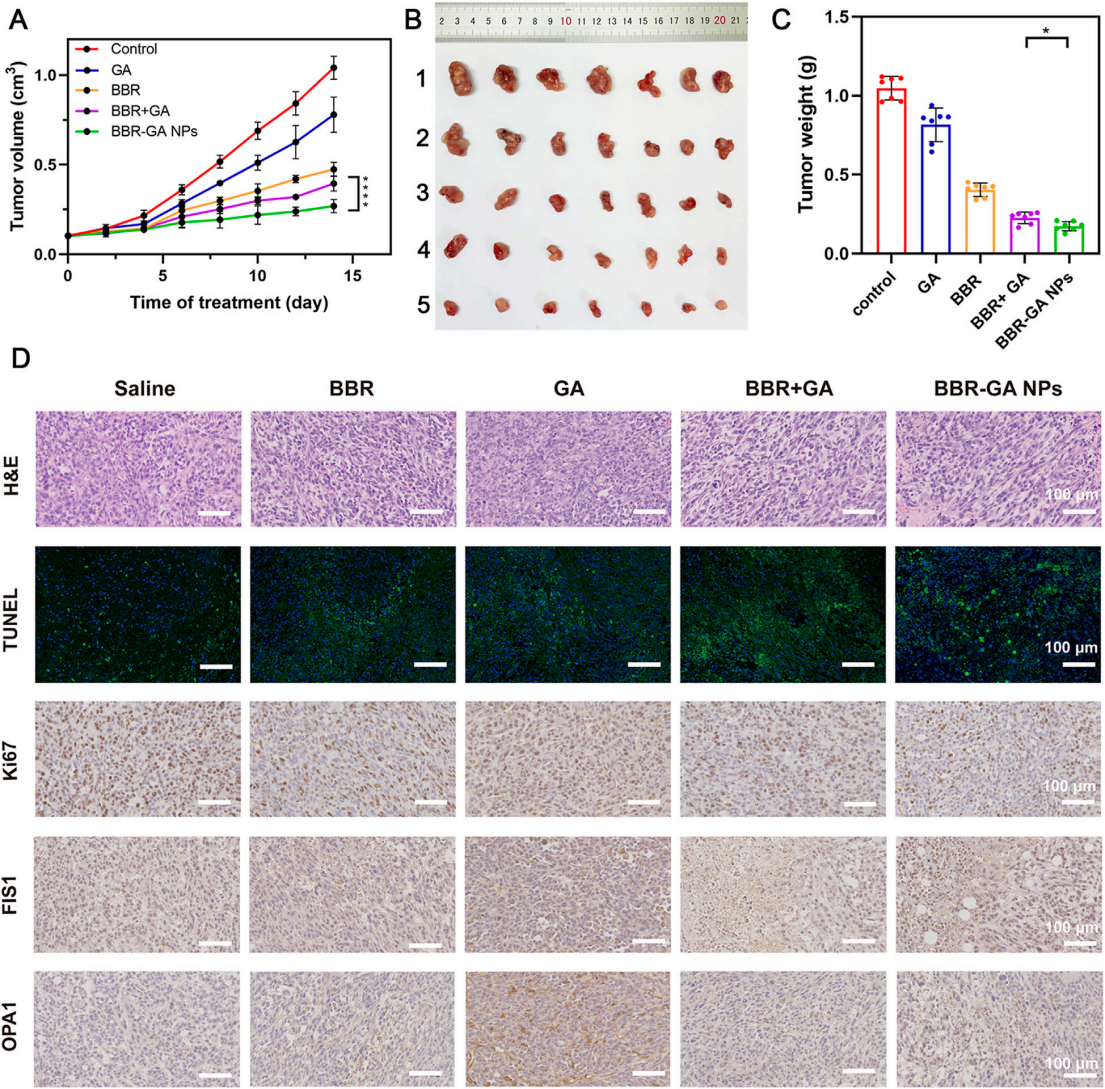
Therapeutic efficacy of BBR-GA NPs in tumor-bearing nude mice *in vivo*. **(A)** Tumor growth curves over 14 days (*n* = 7). ****p < 0.0001. **(B)** Photographs of tumor tissues excised on Day 14. 1: Control; 2: GA; 3: BBR; 4: BBR + GA; 5: BBR-GA NPs. **(C)** Weights of tumor tissues excised on Day 14 (*n* = 7). *p < 0.05. **(D)** Images of H&E staining, TUNEL staining, Ki67, FIS1, and OPA1 staining in tumor slices after treatment.

After verifying their outstanding tumor targeting efficiency in a zebrafish model, the antitumor efficacy of BBR-based NPs was tested in tumor cell-bearing nude mice. *In vivo* antitumor therapy with BBR-GA NPs was investigated in A549 cell-bearing BALB/c nude mice when the tumor size reached 100 mm^3^. The mice were randomly allocated into five groups and intravenously injected with saline, GA, BBR, BBR + GA, or BBR-GA NPs. During treatment, the tumor volume was recorded. The mice treated with BBR-GA NPs exhibited better inhibition of tumor growth than the GA, BBR, and BBR + GA groups throughout the treatment period (Figure 7A). Tissues were harvested after mice were sacrificed. Treatment with BBR-GA NPs resulted in significant inhibition of tumor growth compared to that of the other treatments (Figure 7B). Notably, BBR-GA NPs had a better inhibitory effect than simple monomer mixture. On Day 14 of treatment, the tumor weights of the BBR-GA NPs group were 0.1-, 0.2-, 0.5- and 0.8-fold lower than those of saline, GA, BBR, and BBR + GA groups, respectively (Figure 7C), and the tumor inhibition rates of BBR-GA NPs, BBR + GA, BBR, and GA groups were 72%, 61%, 53%, and 22%, respectively (Supplementary Figure S13). Next, histological assessments of tissues were performed via H&E staining. The different treatment groups exhibited more loosely packed cells and various degrees of necrosis in tumor tissue sections (Figure 7D). For BBR-GA NPs, tumor cells with a high nucleus-to-plasma ratio exhibited diffuse distribution. The nuclei were heteromorphic and cell boundaries were blurry. Tumor cells exhibited necrosis/apoptosis, and the tumor cell death rate was about 60%. In addition, to confirm the inhibition of apoptosis and cell proliferation, TUNEL and Ki-67 immunohistochemistry were used to evaluate the number of apoptotic cells and proliferating cells in tumor tissue sections. The apoptosis rate of the BBR-GA NPs group was 48.0% as compared with the control group. The apoptosis rates of GA, BBR, and BBR + GA groups were 0.30, 0.2, and 0.6 times lower than that of BBR-GA group. Similarly, the number of Ki-67-positive tumor cells in BBR-GA NPs group were 0.4, 0.6, and 0.7 times lower than that in GA, BBR, and BBR + GA groups, respectively. Next, the expression of two key proteins that regulate mitochondrial homeostasis in tumor tissue were also investigated. Compared to other administration groups, FIS1 and OPA1 in BBR-GA NPs group were obviously highly expressed and expressed at low levels, respectively. In addition to the therapeutic effects, the safety of BBR-GA NPs was also evaluated. The body weights of the mice in all groups increased with no significant differences among groups (Supplementary Figure S14). H&E staining revealed that the cells in main organs exhibited no serious damage among the groups (Supplementary Figure S15). Furthermore, BBR-GA NPs did not induce hepatic or kidney toxicity (Supplementary Figure S16).

**FIGURE 8:**
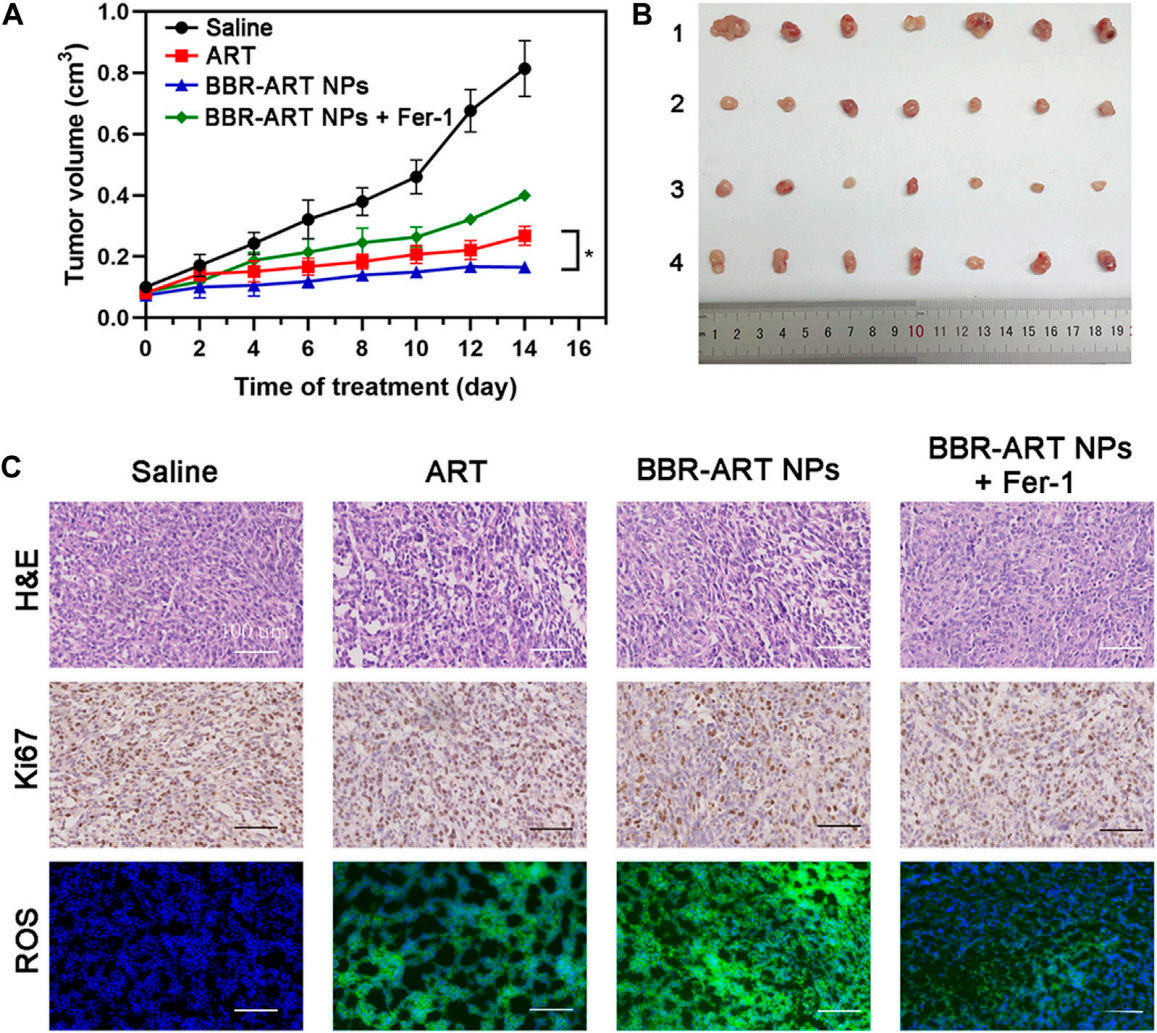
Therapeutic efficacy of BBR-ART NPs in PANC-1 tumor-bearing nude mice *in vivo*. **(A)** Tumor growth curves over 14 days (*n* = 7). *p < 0.05. **(B)** Photographs of tumor tissues excised on Day 14. 1: Control; 2: ART; 3: BBR-ART NPs; 4: BBR-ART NPs + Fer-1; **(C)** Images of H&E staining, TUNEL staining, Ki67, and ROS degree in tumor slices after treatments.

Moreover, *in vivo* antitumor effects of BBR-ART NPs were studied in PANC-1 cell-bearing BALB/c nude mice. The mice were randomly allocated to four groups, and the mice were intravenously injected with saline, ART, BBR-ART NPs, or BBR-ART NPs + Fer-1. BBR-ART NPs-treated group exhibited the lowest tumor volume among all the groups during treatment (Figures 8A, B). Similarly, BBR-ART NPs administration group had the lowest tumor weight among all the groups. The average tumor inhibition rates of ART, BBR-ART NPs, and BBR-ART NPs + Fer-1 groups were approximately 69%, 80%, and 51%, respectively, after 14 days treatment (Supplementary Figure S17). As expected, the addition of Fer-1, a ferroptosis inhibitor, hindered the antitumor efficacy of the BBR-ART NPs. H&E staining was further used to analyze the tumor tissues. BBR-ART NPs produced more loosely packed cells and led to the highest amount of tumor cell death (Figure 8C). Ki-67 immunohistochemistry showed cell proliferation in tumor tissues of the BBR-ART NPs, BBR-ART NPs + Fer-1, and ART groups were 0.3, 0.6, and 0.5 times higher than that of in control group. In addition, compared with those in the PBS group, the BBR-ART NPs group exhibited the maximum ROS level (88.50%). The above research results proved the efficient *in vivo* antitumor activity of BBR-ART NPs. The biosafety of BBR-ART NPs was also evaluated. The body weight of each group showed slight increase, and the difference between groups could be ignored (Supplementary Figure S18). According to the H&E staining results, no serious damage was observed in the main organs among the four groups (Supplementary Figure S19). In addition, compared with those in the control group, BBR-ART NPs did not induce obvious hepatic or kidney toxicity, for example, the blood BUN/CRE (a kidney function marker) and GOT/GPT (liver function marker) levels were normal (Supplementary Figure S20). These results suggested that BBR-ART NPs had good biosafety and did not induce severe systemic toxicity. Thus, BBR-based NPs were proven to have a satisfactory safety profile for synergistic drug delivery and enhanced antitumor efficacy *in vivo*.

## 4 Conclusion

In summary, we developed a carrier-free BBR-based nanoplatform for synergistic tumor treatment. Specifically, BBR can interact with GA or ART to self-assemble BBR-GA or BBR-ART NPs, respectively. The formation of carrier-free self-assemblies were mainly governed by electrostatic and hydrophobic interactions. The diameters of the BBR-ART NPs were larger than those of the BBR-GA NPs indicating different formation processes for these two BBR-based NPs: the parallel conformation between BBR and GA, and the staggered conformation between BBR and ART. After intravenous injection, the assembled BBR-GA NPs accumulated at tumor sites owing to passive targeting effect (EPR). BBR and GA were released into cytosol, after which BBR specially targets the mitochondria in tumor cells. BBR-GA NPs could lead to mitochondria-mediated cell apoptosis by regulating mitochondrial fission and dysfunction. Moreover, *in vivo* and *in vitro* trials showed that BBR-ART NPs could induce ferroptosis in PANC-1 cells. Therefore, BBR-based NPs enabled synergistic antitumor and sensibilized single drug-based antineoplastic effect. These carrier-free self-assemblies based on natural products provide a strategy for synergistic drug delivery and thus offer broad prospects for developing enhanced antitumor drugs. The synergistic strategy we constructed is a promising candidate for clinical application in the treatment of tumors in the future.

## Data Availability

The original contributions presented in the study are included in the article/Supplementary Material, further inquiries can be directed to the corresponding authors.
