# Supplementary material for: Berberine-based self-assembly agents with enhanced synergistic antitumor efficacy
Source: Front Pharmacol. 2024 Mar 12;15:1333087. doi: 10.3389/fphar.2024.1333087 (PMC10967022; doi:10.3389/fphar.2024.1333087)
Supplement: Supplementary file 1 [file Presentation1.pdf]

Supporting Information

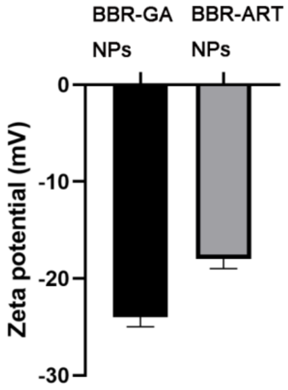

Fig. S1 zeta potentials of BBR-based NPs. Data were mean ± SD, n = 3.

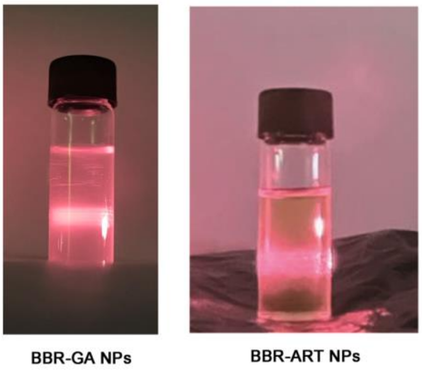

Fig. S2 Tyndall effect in aqueous solution of BBR-based NPs.

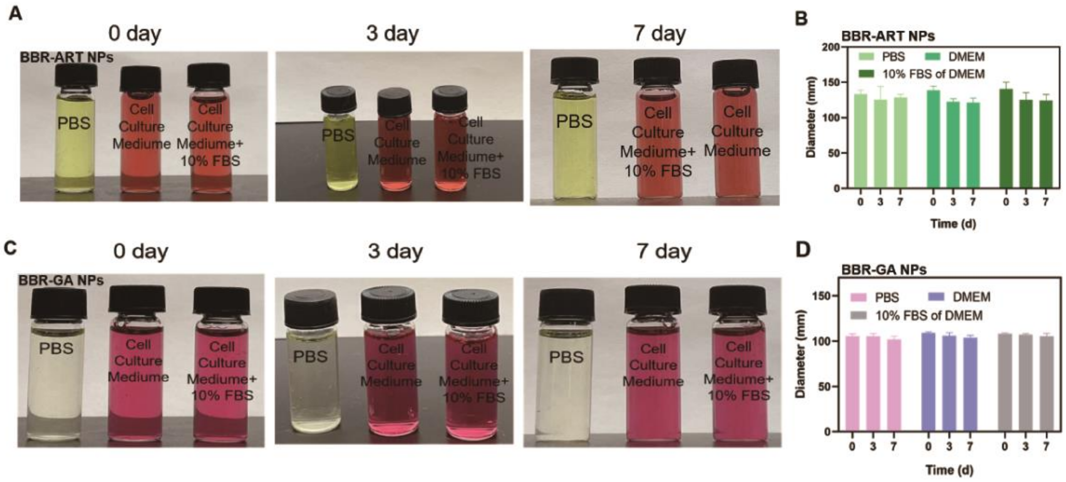

Fig.S3 Stability of BBR-based NPs. (A, C) The BBR- based NPs in PBS, DMEM and DMEM (10% FBS) after 3 and 7 d incubation. (B, D) Particle size variation of BBR-based NPs in PBS, DMEM, and DMEM (10% FBS) after 3 or 7 days of incubation. Data were mean ± SD, n = 3.

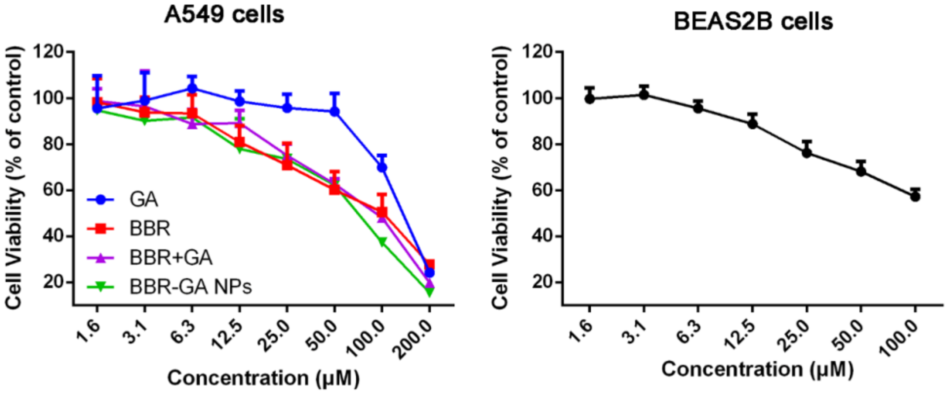

Fig. S4 MTT assays of A549 and BEAS-2B cells after incubation with GA, BBR, BBR + GA and BBR-GA NPs for 48 h. Error bars indicate the SD (n = 3).

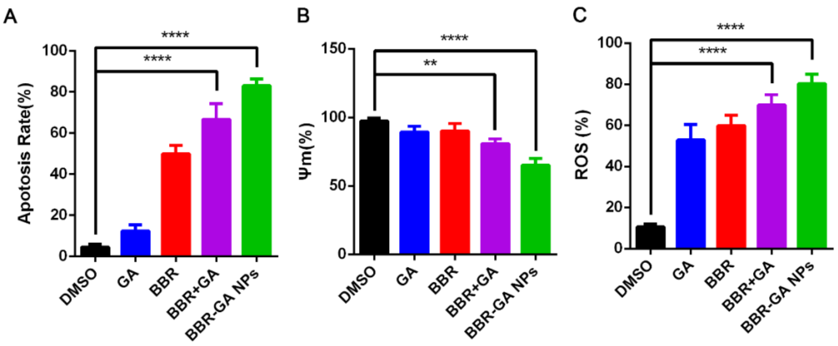

Fig. S5 Apoptosis rate (A), mitochondrial membrane potential (ΔΨm) (B) and intracellular ROS generation (C) of A549 cells following various treatments. Error bars indicate the SD (n = 3). $^{**}p < 0.01$ and $^{****}p < 0.0001$.

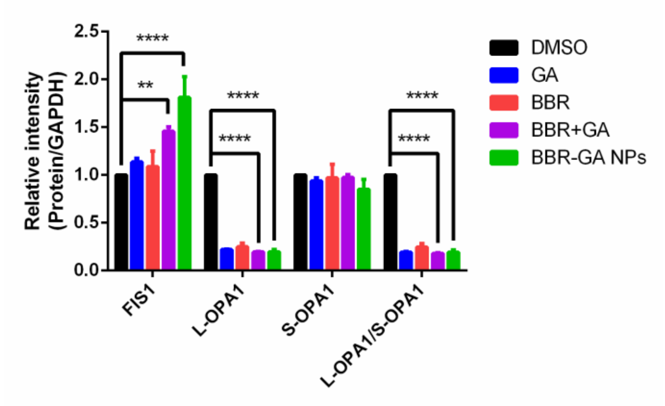

Fig. S6 The levels of FIS1, L-OPA1, and L-OPA1 of A549 cells treated with various formulations for 48 h by Western blotting. Error bars indicate the SD (n = 3). $^{**}p < 0.01$ and $^{****}p < 0.0001$.

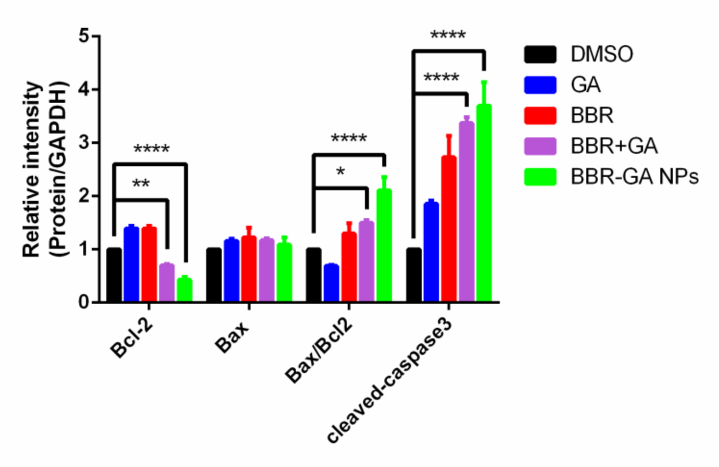

Fig. S7 The levels of Bcl-2, Bax, and cleaved caspase3 of A549 cells treated with various formulations for 48 h by Western blotting.. Error bars indicate the SD (n = 3). $^{*}p < 0.05$, $^{**}p < 0.01$ and $^{****}p < 0.0001$.

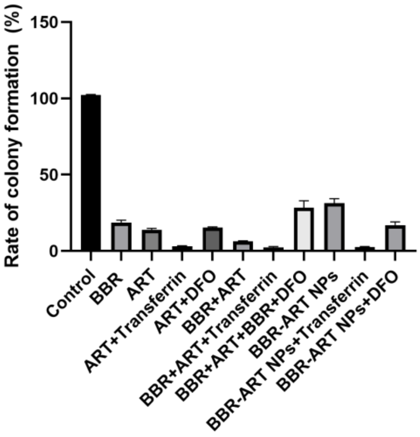

Fig. S8 Colony formation rate of PANC-1 cells treated with various formulations in colony formation assays. Error bars indicate the SD (n = 3).

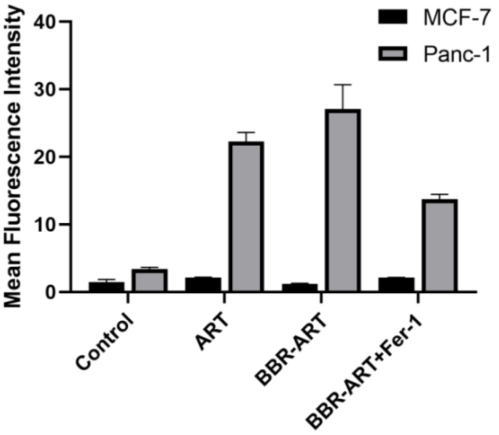

Fig. S9 Mean fluorescence intensity of MCF-7 and PANC-1 cells treated with various formulations by HCS system. Error bars indicate the SD (n = 3).

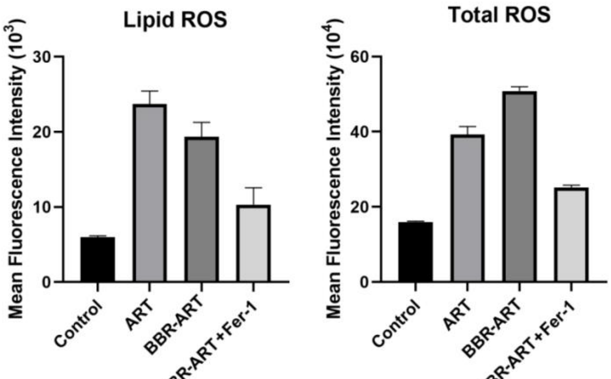

Fig. S10 The levels of lipid ROS and total ROS generation of PANC-1 cells following various treatments. Error bars indicate the SD (n = 3). $^{**}p < 0.01$ and $^{****}p < 0.0001$.

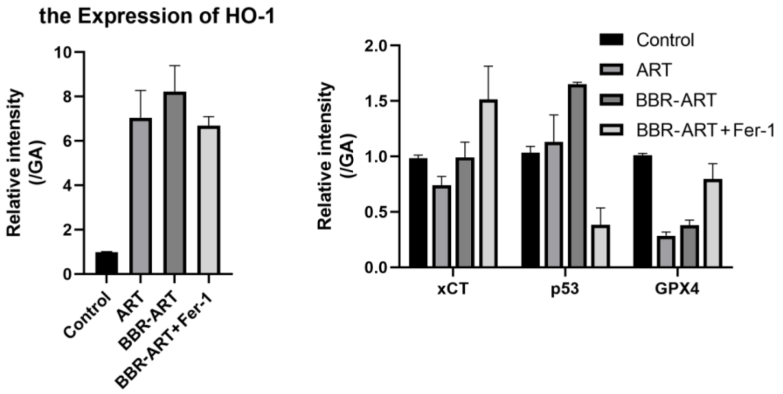

Fig. S11 The levels of HO-1, xCT, p53, and GPX4 of PANC-1 cells treated with various formulations for 24 h by Western blotting. Error bars indicate the SD (n = 3).

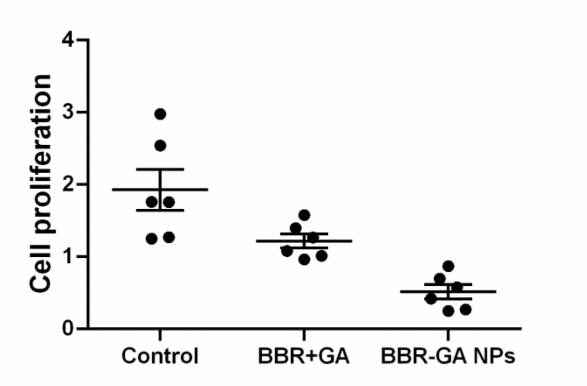

Fig. S12 Cell proliferation of A549 tumor-bearing zebrafish treated with various formulations (n = 6).

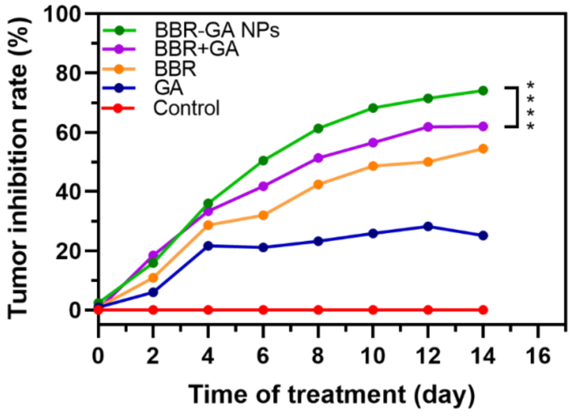

Fig. S13 Tumor inhibition rate curves over 14 days during various treatments (n = 7). $^{****}p < 0.0001$.

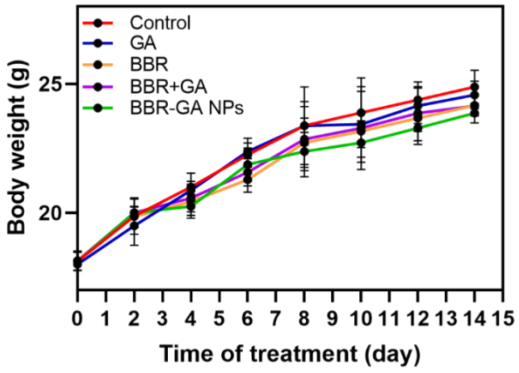

Fig. S14 Body weight curves over 14 days during various treatments (n = 7).

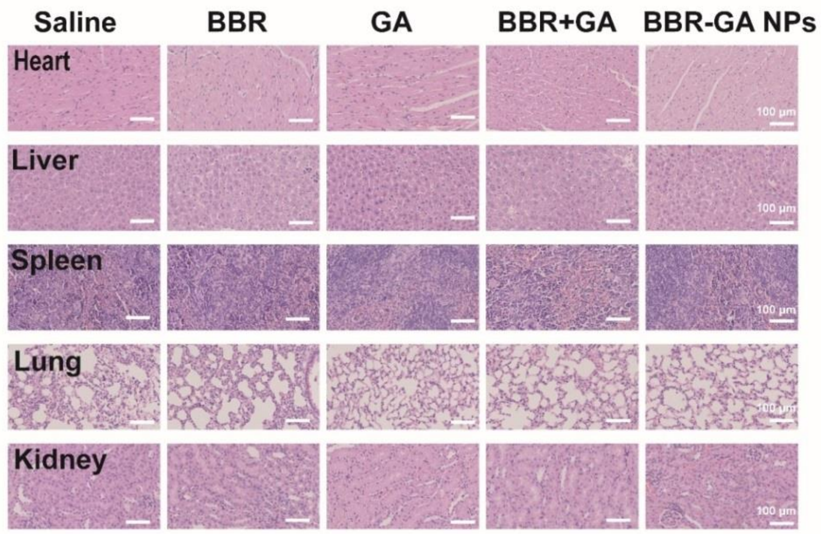

Fig. S15 Histological observation of major organs of various treatments by H&E. Scale bars: 100 μm.

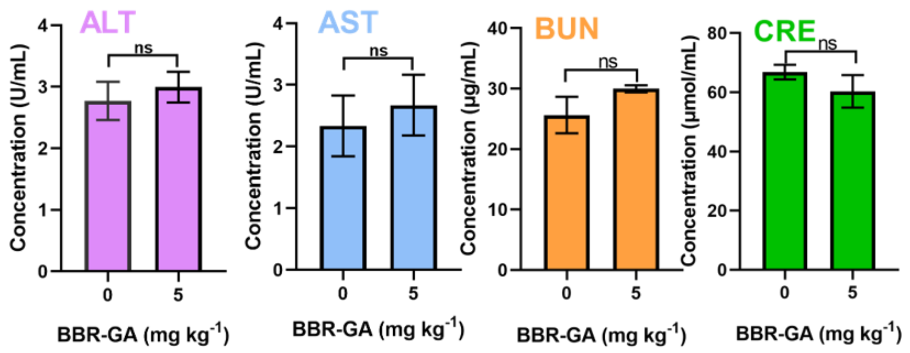

Fig. S16 Evaluation of liver and renal functions after various treatments. Data were mean ± SD, n = 3.

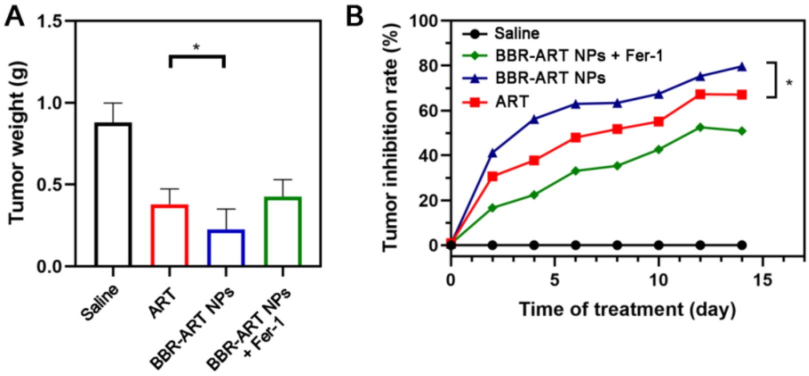

Fig. S17 (A) Weights of tumor tissues excised on Day 14 following various treatments (n = 7). $^{*}p < 0.05$. (B) Tumor inhibition rate curves over 14 days during various treatments (n = 7). $^{*}p < 0.05$.

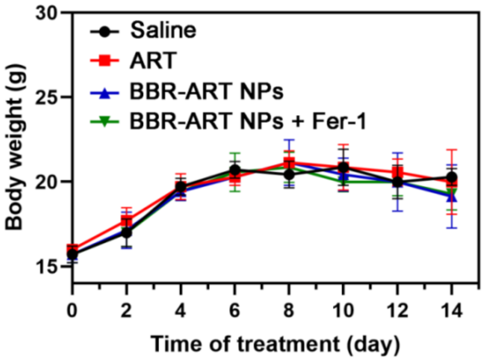

Fig. S18 Body weight curves over 14 days during various treatments (n = 7).

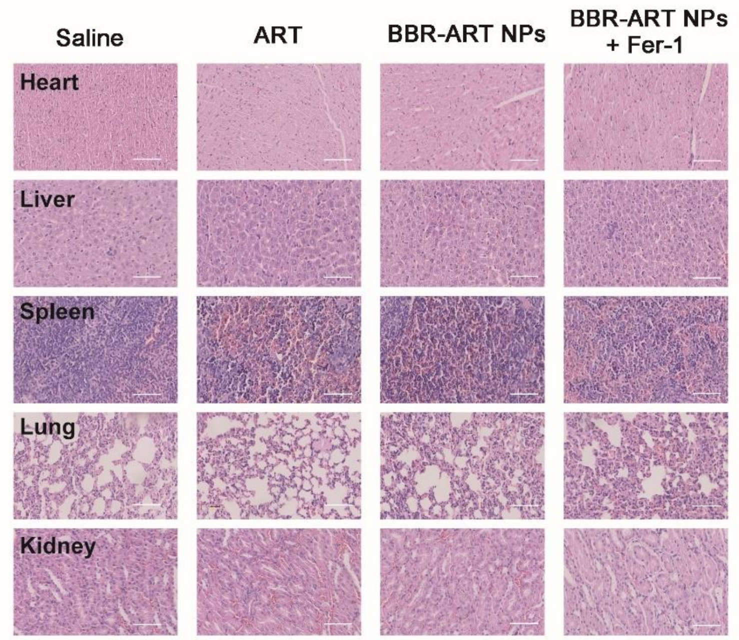

Fig. S19 Histological observation of major organs of various treatments by H&E. Scale bars: 100 μm.

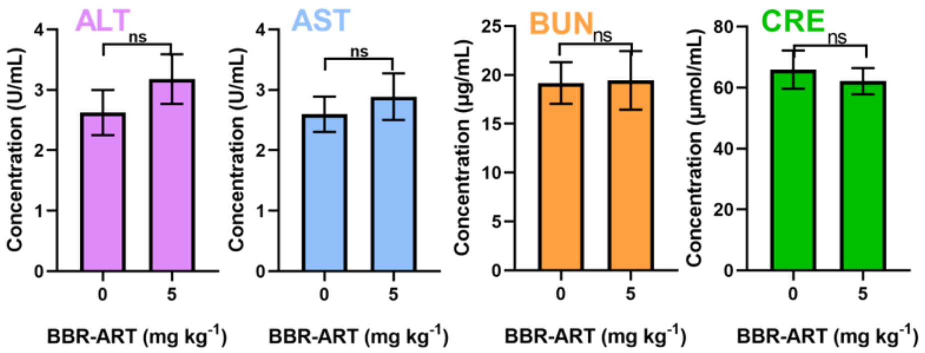

Fig. S20 Evaluation of liver and renal functions after various treatments. Data were mean ± SD, n = 3.
